# Effect of Stacking Sequence on Fatigue Performance of CFRP–Al Single-Lap Adhesive Joints: Experimental Study

**DOI:** 10.3390/polym14235088

**Published:** 2022-11-23

**Authors:** Tianchun Zou, Yuezhang Ju, Yuxi Guan, Ji Fu

**Affiliations:** College of Safety Science and Engineering, Civil Aviation University of China, Tianjin 300300, China

**Keywords:** CFRP–Al single-lap joints, stacking sequence, fatigue performance, failure mechanisms

## Abstract

This study aimed to explore failure mechanisms of carbon fibre-reinforced plastic (CFRP)–aluminium (Al) single-lap adhesive joints which CFRP adherends had different stacking sequences. These results showed that fatigue performance of CFRP decreased as the number of 45° plies increased, which caused the initial failure location to gradually move from the adhesive layer towards the CFRP. Under high load levels, joint-failure models were influenced by the stacking sequence of CFRP; large-area cohesive failure occurred in joints when the CFRP stacking sequence was [0/90]_4s_ and [0/45/−45/90]_2s_, and delamination failure occurred when the CFRP stacking sequence was [45/−45]_4s_, due to the weak interlaminar properties of CFRP. However, under low load levels, the stacking sequence of CFRP had little effect on the failure model of the joint, with interfacial failure being the main failure mode for all joints due to weakening of the mechanical interlock.

## 1. Introduction

Employing lightweight materials to reduce structure weight is one of the most effective ways to solve the problems of rising carbon emissions and environmental degradation [1,2,3,4]. Carbon fibre-reinforced plastic (CFRP) is a material with exceptional properties such as high strength, light weight, and corrosion resistance. It has been extensively used in the building, aerospace, and automotive industries to solve problems related to structure repair [5,6,7] and reinforcement [8,9,10,11,12,13] or to form new structures for engineering applications [14].

However, in practical engineering applications of composite connections, composite materials cannot completely replace metal materials, so the connection between CFRP and Al has become a hot topic [15]. Compared with traditional connection methods (bolting and riveting), adhesive technology is widely adopted in wings, fuselages, and multi-materialized vehicle bodies due to its great structural integrity and rational design flexibility [16,17,18,19]. Numerous factors affect mechanical properties of CFRP adhesive joints, such as stacking sequence, adhesive thickness, and overlap length. [20,21,22,23,24,25]. Among these, stacking sequence can change the mechanical properties of the composite by altering interlaminar stress, which provides a reference for the design of braided CFRP connection structures [25]. Therefore, many scholars are focused on the study of the effect of stacking sequence on mechanical properties of joints.

Hitherto, Jiang et al. [20] investigated quasi-static failure behaviour of CFRP–CFRP adhesive joints. They revealed that the direction of a fibre contact with adhesive has a significant influence on overall structural strength. Compared with the ±45 plies, when fibre contact with the adhesive is 0/90 plies, the joint had greater strength. The numerical and experimental studies of Ozel et al. [21] explored how CFRP adherends with different stacking sequences significantly affect joint-failure load and stress distribution. The results of that study showed that stresses formed at the ends of the overlap region gradually increased when the stacking sequence of the CFRP adherend changed from [0/90]_8_ to [0/45/−45/90]_4_, then to [45/−45]_8_. Zhang et al. [22] studied the tensile performances of adhesive joints by designing three stacking sequences ([45/90_2_/−45/90_2_/45/0/−45/90/45/0/−45/90/45/0_2_/−45/0_2_], [45/0/−45/90/45/0/−45/90/45/0]_s_ and [0]_20_) for CFRP. They found that stress distribution between different layers could influence failure models of joints. Under the same applied load, [0]_20_ had the least Mises stress, leaving the smallest area for cohesive failure.

Many studies have focused on effects of the stacking sequence on quasi-static mechanical properties of adhesive joints. However, during long-term service, structures experience cyclic loads that result in 50–90% of mechanical failures and threaten their service life severely [26,27]. At the same time, owing to excellent sealing performance of the adhesive, it is difficult to check for internal failure of an adhesive joint in time. This can result in damage accumulation and overall structure destruction [28]. Therefore, fatigue resistance of adhesive joints is an essential property in connecting CFRP and Al [29,30]. In this regard, Mariam M. et al. [31] investigated tensile fatigue properties of aluminium alloy (AA7075) and GFRP single-lap adhesive joints. Their results indicated that fatigue damage mechanisms in composites were considerably more complicated than those in metal adherends. Fibre breakage, extensive delamination, and matrix cracks led to composite failure, as metal surfaces only experienced mixed mode and adhesive failure. S. Azari et al. [32] investigated the effect of adherend thickness on the fatigue performance of joints under cyclic loads. They revealed that due to greater global stiffness and larger crack-tip stresses of thicker joints, fatigue properties of joints decreased when adherend thickness (h) increased, and when h > 12.7 mm, joint fatigue behaviour was independent of h. Kara E et al. [33] studied the effect of overlap length on static and fatigue behaviours of adhesive joints. They found that increasing overlap length increased static strength but decreased fatigue life. Shin et al. [34] investigated the effect of the stacking sequence on fatigue performance of the steel-composite co-cured joint. They found that under a high load level, a joint with a composite stacking sequence of [±45]_4s_ had more transverse shear stress than a joint with the [0]_16_ condition, indicating that the [0]_16_ sample had good fatigue characteristics. R Hedayati et al. [35] compared tensile fatigue properties of Al-composite adhesive joints with composite adherend stacking sequences of [0/90]_2s_ and [0/45/−45/90]_s_. Their results indicated that cases with a stacking sequence of [0/45/−45/90]_s_ had much a shorter fatigue life than cases with [0/90]_2s_ because using a composite with a stacking sequence of [0/45/−45/90]_s_ transferred more stress to the adhesive crack front in comparison with the cases with [0/90]_2s_. G. Meneghetti et al. [36] performed an experimental study of failure mechanisms in adhesive joints that composited adherends with different stacking sequences under cyclic loads. They revealed that compared with 0° plies, presence of 45° plies could slightly extend crack propagation life, but that effect tended to disappear when total fatigue life was considered.

To sum up, compared with influencing factors such as overlap length, adhesive thickness, etc., there are fewer studies on effects of stacking sequences on fatigue performance of adhesive joints, and most mainly focus on the influence of joint fatigue life. Meanwhile, limited attempts have been made to reveal failure mechanisms of joints under cyclic loads.

In this paper, fatigue performance and the failure mechanisms of CFRP–Al single-lap adhesive joints that CFRP adherends with different stacking sequences were studied. For this purpose, first, CFRP laminates with three typical stacking sequences ([0/90]_4s_, [0/45/−45/90]_2s_, and [45/−45]_4s_ (where subscript number n indicates repetition of n plies and subscript s indicates symmetrical arrangement of plies) were selected to fabricate adhesive joints, and a series of quasi-static and fatigue tests was carried out for those joints. Second, based on experimental data and the Weibull method, joint mean fatigue life was calculated and SN curves were fitted using multiple function models. Finally, microscopic fracture morphologies were measured by SEM in order to analyse differences in failure modes of the joints, thereby further revealing failure mechanisms of the joints under cyclic loads.

## 2. Materials and Methods

### 2.1. Materials

CFRP laminates with three typical stacking sequences ([0/90]_4s_, [0/45/−45/90]_2s_, and [45/−45]_4s_) and aluminium 7075-T651 plates were selected as adherends, as they are commonly employed in the aviation sector. The CFRP laminates were fabricated using carbon fibre/epoxy prepreg (USN15000/7901/RC33, CETC Wuhu Diamond Aircraft Manufacture Co., Ltd., Wuhu, China) via autoclave moulding technology. Mechanical properties of the USN15000/7901/RC33 and aluminium are shown in Table 1 and Table 2, respectively. Furthermore, Araldite 2015 (Huntsman, SLC, US)), a two-component epoxy resin adhesive that cures at room temperature, was used to bond the adherends.

To simplify expression of the joints which CFRP with different stacking sequences, the specimens are abbreviated as J-stacking sequences, as shown in Table 3.

### 2.2. Preparation of Specimens

**Figure 1:**
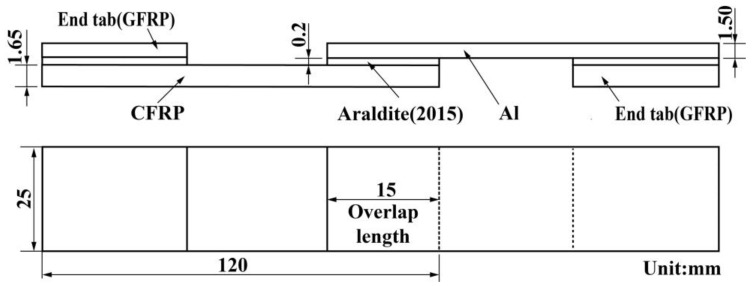
Geometry of CFRP–Al single-lap adhesive joints.

According to the standard of ASTM D3166 [39], the CFRP laminates were cut into rectangular plates with a size of 120 × 25 × 1.65 mm each. Similarly, Al was cut into plates with dimensions of 120 × 25 × 1.5 mm for joints. The joint overlap length and adhesive thickness were designed to be 15 mm and 0.2 mm, respectively, and end tabs with the same thickness as the adherends were bonded at the free ends of each joint to make the joint better align with the gripper during the fatigue test process. Specific structural parameters are shown in Figure 1.

Before bonding of specimens, waterproof abrasive papers (80 mesh) were used to polish each adherend bonding surface in order to increase its roughness. During the polishing process, polishing direction, polishing intensity, and polishing frequency were kept consistent for all specimens. Acetone was subsequently used to remove detritus and oil contamination from each adherend surface.

**Figure 2:**
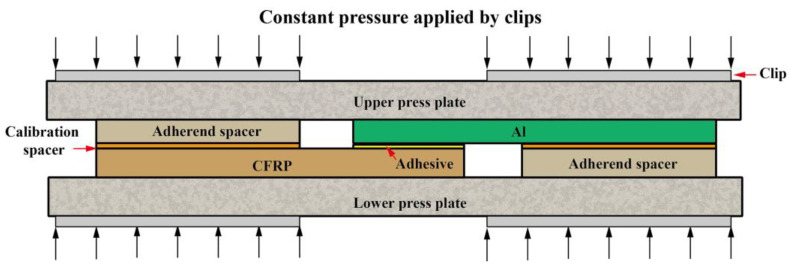
Configuration for fabrication of single-lap joints.

To obtain valid test results, 0.2 mm calibration spacers and adherend spacers with the same thickness as substrates were used to control thickness of the adhesive layer. After fixing of the spacers, the joints were pressurized in 0.6 MPa by the upper and lower pressure plates and cured at room temperature. The schematic diagram of the control method of adhesive thickness is shown in Figure 2.

### 2.3. Test Instruments

#### 2.3.1. Quasi-Static Tensile Test

**Figure 3:**
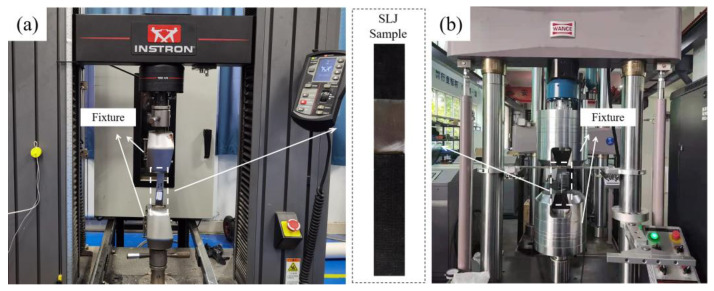
Testing equipment: (**a**) universal testing machine and (**b**) electro-hydraulic servo fatigue testing machine.

Quasi-static tensile tests were conducted in a universal testing machine (Instron 9520, USA) equipped with a 100 kN load cell, as shown in Figure 3a. The joints were stretched at a constant speed of 2 mm/min in accordance with ASTM D5868-01 [40]. The Al adherend was fixed and the joint was stretched at the end of the CFRP. Failure loads and displacements of the joints were recorded.

#### 2.3.2. Fatigue Test

The electro-hydraulic servo fatigue testing machine (100 kN) was used to conduct fatigue tests of the joints under different load levels, as shown in Figure 3b. In the fatigue test, the Al adherend was fixed and the joint was cyclically stretched at the end of the CFRP in the same way as in the quasi-static tensile test. According to the experimental results from S. Çavdar et al. [41], when load levels reached 80% of peak load, specimens will immediately fracture, making data collection extremely challenging. Therefore, load levels were reduced from 75% of peak load, and four different load levels were applied to obtain effective fatigue life of the joints (less than 10^6^ cycles) for analysis. According to the standard of ASTM D3166 and the numerous literatures that study the fatigue behaviour of Araldite 2015, fatigue tests were carried out under 30 Hz [39,42,43,44]. The load ratio was set to 0.1, and four samples were tested under each load level to ensure that the test results were repeatable and reliable.

#### 2.3.3. Fatigue Analysis

Weibull distribution, normal distribution, and logarithmic normal distribution are often used as statistical analysis methods to describe the distribution law of test data from the fatigue test [45]. Compared with other methods, Weibull distribution is more extensively applied. In this study, two-parameter Weibull distribution was adopted to deal with joint fatigue life. Probability density and cumulative distribution functions (namely reliability) in the Weibull model can be represented as [43,46]:

(1) $$D\left(t\right)=\frac{\alpha }{\beta }{\left(\frac{t}{\beta }\right)}^{\alpha -1}e^{-{\left(\frac{t}{\beta }\right)}^{\alpha }}$$

(2) $$F\left(t\right)=e^{-{\left(\frac{t}{\beta }\right)}^{\alpha }}$$

where *t* is a random variable that refers to the joint fatigue life, *α* is shape parameters, and *β* is scale parameters.

Based on Equation (1), reliability *R*(*t*) can be estimated as

(3) $$R\left(t\right)=e^{-{\left(\frac{t}{\beta }\right)}^{\alpha }}$$

Taking the logarithm of both sides of Equation (3) can obtain the following formula:

(4) $$\ln\ln\frac{1}{R\left(t\right)}=\alpha \ln\left(t\right)-\alpha \ln\left(\beta \right)$$

It can be seen from Equation (4) that lnln [1/*R*(*t*)] has a linear relationship with ln(*t*), and *α* and *β* can be obtained through the intercept. However, *F*(*t*) cannot be calculated through Equation (2), so the definition replaces reliability in order to describe reliability as

(5) $$\overset{\cdot }{R}\left(t\right)=1-\frac{i-0.3}{n+0.4}$$

where *i* is the serial number of the specimen under a particular load level and *n* is the total number of fatigue samples under a specific load level.

Shape parameters *α* and scale parameters *β* could be calculated through Equation (4), and the mean life (mean time to fatigue, or *MTTF*) of joints was determined as

(6) $$MTTF=\int_{0}^{+\infty }tF\left(t\right)dt=\beta \;\Gamma \left(1+\frac{1}{\alpha }\right)$$

To evaluate relative dispersion of fatigue life under different load levels, the standard deviation (*SD*) and coefficient of variation (*CV*) were used for respective assessment:

(7) $$SD=\sqrt{\beta^{2}\left[\Gamma \left(1+\frac{2}{\alpha }\right)-\Gamma^{2}\left(1+\frac{1}{\alpha }\right)\right]}$$

(8) $$CV=\frac{SD}{T}=\frac{\sqrt{\Gamma \left(1+\frac{2}{\alpha }\right)-\Gamma^{2}\left(1+\frac{1}{\alpha }\right)}}{\Gamma \left(1+\frac{1}{\alpha }\right)}$$

where Γ is the gamma function [47].

## 3. Results and Discussion

### 3.1. Quasi-Static Tensile Behaviour

**Figure 4:**
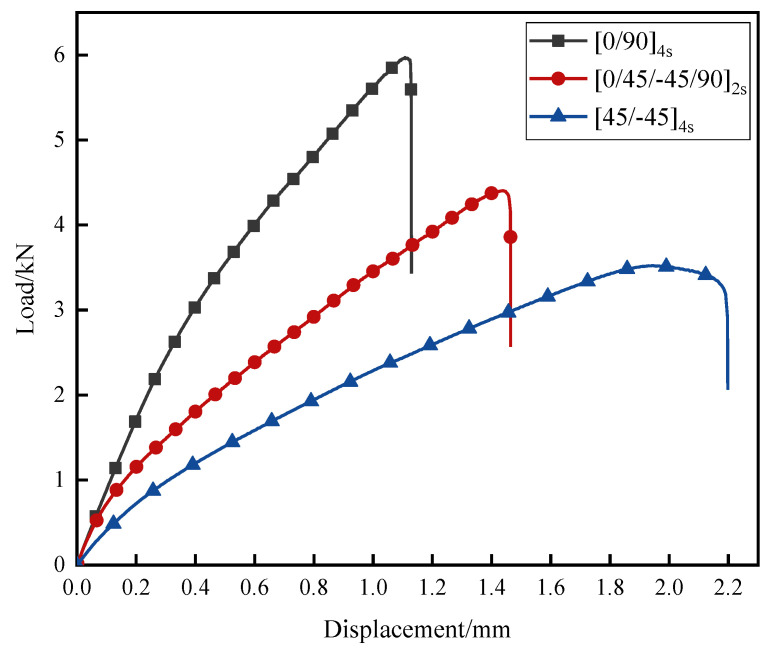
Typical load–displacement curves of the joints.

Figure 4 presents typical tensile load–displacement curves of the joints, while values of joint-failure load and displacement are listed in Table 4. It can be seen that J-[0/90]_4s_ had a maximum failure load of 5.93 kN and J-[45/-45]_4s_ had a minimum failure load of 3.52 kN. It can also be clearly seen that from J-[0/90]_4s_ to J-[0/45/-45/90]_2s_, then to J-[45/-45]_4s_, the failure loads of the joints decreased gradually. Since the fibres bear the main load in CFRP, it can be presumed that the more 0° plies are in the same direction as the load, the higher the failure load of the joints will be. Meanwhile, it was found that J-[45/-45]_4s_ exhibited a plastic deformation stage during the test; that is, the load continued at around 3.5 kN, but joint displacement increased from 1.82 mm to 2.08 mm, while J-[0/90]_4s_ and J-[0/45/-45/90]_2s_ exhibited a linear elastic stage until a brittle fracture occurred. The main reason for this is that ±45 plies can rotate and deform in the loading direction when the ply angle is changed. Along with tensile stress, the matrix is subjected to compression stress caused by deformation, and eventually, shear or crush failure will occur [48]. The matrix will lose support and restraint of the CFRP adherend, thereby reducing bearing capacity of the CFRP adherend and causing more deformation [49].

### 3.2. Fatigue Life

Two-parameter Weibull distribution was used to analyse fatigue life of the joints, and SN curves were performed to explore the characterization function suitable for joint fatigue life. Table 5 shows detailed fatigue life and calculation results of lnln [1/*R*(*t*)] and ln(*t*). Considering fatigue life of the joints, it can be found that, from J-[45/−45]_4s_ to J-[0/45/−45/90]_2s_ and then to J-[0/90]_4s_, the load level which joint fatigue life exceeding 10^6^ cycles increased from 15% to 25% to 35%, respectively. Cycles greater than 10^6^ were generally considered to be the infinite cycle of each joint, which meant that no failure would be experienced when a cycle was greater than 10^6^. There was an indication that as the number of 45° plies increased, the joints became more resistant to damage.

**Figure 5:**
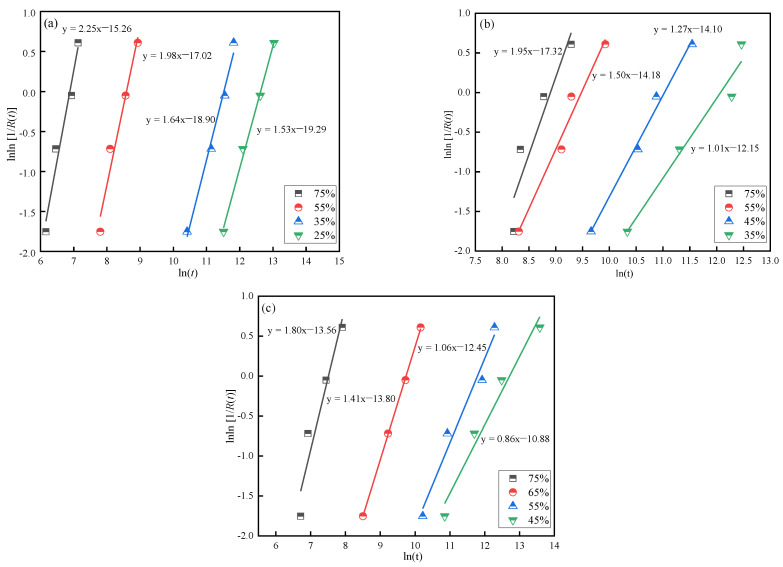
Weibull probability plot under different load levels: (**a**) [0/90]_4s_, (**b**) [0/45/−45/90]_2s_, and (**c**) [45/−45]_4s_.

Equation (4) shows a linear relationship between lnln [1/*R*(*t*)] and ln(*t*). Linear fitting was performed to obtain the shape parameter (*α*) and scale parameter (*β*) of the Weibull distribution mathematical model, as shown in Figure 5. Based on Equations (6)–(8), *MTTF* and the *CV*s of the joints are presented in Table 6.

**Figure 6:**
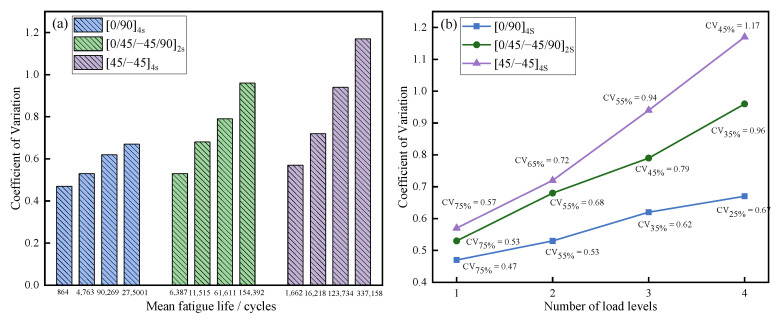
Variations of the *CV* of the joints: (**a**)The relationship between *MTTF* and the *CV,* (**b**)The relationship between load level and the *CV*.

Through analysis of *MTTF* and the *CV*s of the joints under different load levels, it was found that *CV*s showed an upward trend with an increase of *MTTF* (as shown in Figure 6a), indicating that volatility of joint fatigue life rose as load levels decreased and fatigue life reliability of the joints was reduced. At the same time, from J-[45/−45]_4s_ to J-[0/45/−45/90]_2s_, then to J-[0/90]_4s_, the *CV*s of the joints decreased, as shown in Figure 6b, illustrating that fatigue performance stability of the joints decreased when the number of 45° plies increased. The explanation for this phenomenon is that structural strength tends to change uniformly under cyclic loads as a result of involvement of a large number of fibres in the load direction [50]. Therefore, the *CV* of J-[45/−45]_4s_ was larger than that of J-[0/90]_4s_ and J-[0/45/−45/90]_2s_.

**Figure 7:**
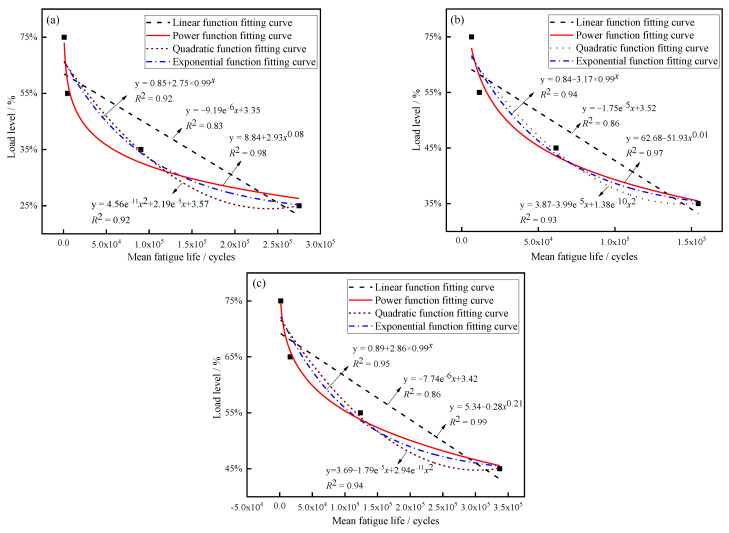
Typical SN curves of the joints: (**a**) [0/90]_4s_, (**b**) [0/45/−45/90]_2s_, and (**c**) [45/−45]_4s_.

Figure 7 shows the SN curves of three kinds of joints under different load levels. According to the relevant results based on various function models, *R*^2^ was the largest when the fitting functions were power functions (0.98 for J-[0/90]_4s_, 0.97 for J-[0/45/−45/90]_4s_, 0.99 for J-[45/−45]_4s_), which means that the highest fit. Function expressions are shown in Equations (9)–(11):

[0/90]_4s_: y = 8.84 + 2.93*x*^0.08^ (9)

[0/45/−45/90]_2s_: y = 62.68 − 51.93*x*^0.01^ (10)

[45/−45]_4s_: y = 5.34 + −0.28*x*^0.21^ (11)

According to the SN curves, the *MTTF* of the joints did not change significantly under high loads, and the *MTTF* gradually increased with a decrease in load levels. This means that joints are more sensitive under low loads, which resulted in the *CV*s of the samples increasing gradually with the decrease in load levels.

### 3.3. Failure Mode

**Figure 8:**
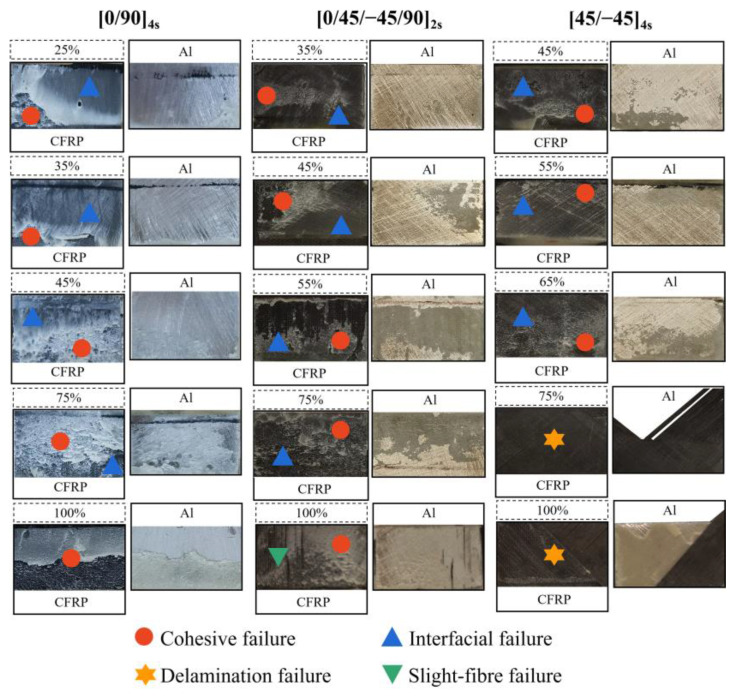
Diagram of failure mode of joints under different load levels.

Figure 8 exhibits the typical failure modes of the joints, while the percentage of area occupied by the different failure modes of the joints is shown in Table 7. With increasing load levels for J-[0/90]_4s_, area of cohesive failure increased, area of interfacial failure decreased, and complete cohesive failure occurred at 100% of the peak load. On one hand, under a high cyclic load, the adhesive defect had a more significant effect on joint failure than did interface defect [51]. On the other hand, due to the larger displacement per cycle at the high load level, crack propagation velocity was higher in the adhesive along the overlap length. These factors resulted in a larger cohesive failure area with increasing load levels. Moreover, under 100% of the failure load, there were a lot of slight cracks in the lap end of the CFRP adherend. This occurred because the lower elastic modulus of the CFRP was the first to deform in the tension process [51].

For J-[0/45/−45/90]_2s_, the variation pattern of the joint-failure model at different load levels was similar to that of J-[0/90]_4s_; with increasing of load levels from 35% to 45% to 55% to 75%, the proportion of cohesive failure areas increased from 20% to 34% to 62% to 85%, respectively. When the load level reached 100% of the failure load, CFRP exhibited slight fibre failure, indicating a weaker performance when the stacking sequence was [0/45/−45/90]_2s_ as opposed to [0/90]_4s_. In general, extension of cracks in the matrix is prevented when there are more intersections between different plies, but this factor was not applicable to this phenomenon, indicating that the number of fibres in the same direction as the cyclic load has a greater effect on joint fatigue performance than the number of intersections between multiple layers [50].

For J-[45/−45]_4s_, with the increase of load levels from 45% to 65%, the proportion of cohesive failure areas increased from 23% to 49%. However, except for in interfacial failure and cohesive failure, delamination failure occurs when load levels reach 75% and 100% of the failure load. On one hand, there were no fibres in the same direction as the cyclic load, causing the matrix to carry the main load. On the other hand, J-[45/−45]_4s_ had weak interlaminar properties at high load level due to fewer intersections between its different layers. Therefore, it was easy for J-[45/−45]_4s_ to delaminate under high loads.

### 3.4. Fracture Analysis

**Figure 9:**
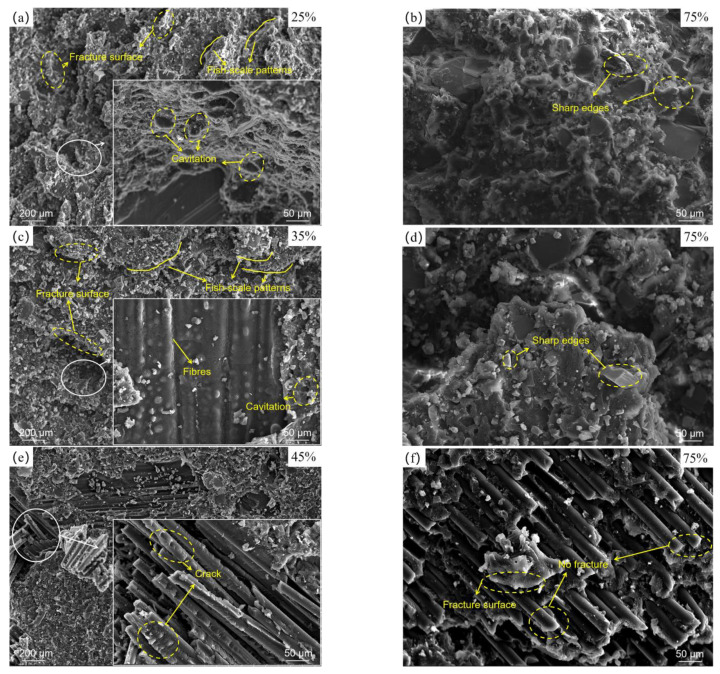
Micro-failure morphology of joints under different load levels: (**a**,**b**) [0/90]_4s_, (**c**,**d**) [0/45/−45/90]_2s_, and (**e**,**f**) [45/−45]_4s_.

Figure 9 shows typical microscopic fracture morphologies of the joints, obtained through SEM. In the enlarged view of Figure 9a,c,e, it can be seen that compared with J-[0/90]_4s_, the failure location of J-[0/45/−45/90]_2s_ was close to the CFRP adherend. For J-[45/−45]_4s_, it could be observed that the matrix partially failed, and some parabolic shape cracks existed on the fibres, revealing that the CFRP adherend was subjected to shear stress [52]. It was shown that the initial failure occurred at the CFRP adherend near the adhesive. This phenomenon indicates that from J-[0/90]_4s_ to J-[0/45/−45/90]_2s_, then to J-[45/−45]_4s_, the location of initial damage gradually moved from the adhesive to the CFRP. The main reason for this is that the location of initial failure was biased toward the weakest part of the joint, and overall fatigue properties of CFRP gradually decreased as the number of 0° plies declined.

**Figure 10:**
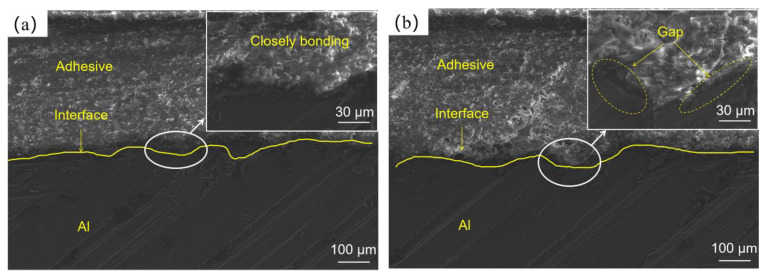
Cross-sections of adhesive joints: (**a**) no cyclic load applied and (**b**) cyclic load applied at 35% load level.

Under the low load level, some holes (Figure 9a,c) in the adhesive could be seen due to thermal expansion of the cavities in the adhesive, indicating that temperature inside the joints had an upward trend after massive cycles [53]. In addition, the Al surface was smooth, without any adhesive residue, as shown in Figure 8, demonstrating that adhesion strength decreased at the low load level: a phenomenon caused by weakening of mechanical interlocking of the interface. The cross-sections of the adhesive joints as shown in Figure 10 could explain the weakening phenomenon of mechanical interlock. Figure 10a shows interface morphology of the Al and of the adhesive layer of the joint not subjected to cyclic load, while Figure 10b displays those of the joint subjected to cyclic load at 35% load level. This image shows that there were almost no gaps at the interface of the joint not subjected to cyclic load, resulting in more compact mechanical locking. However, at low load level, extensive gaps were found at interface, indicating the poor penetration of adhesive into grooves. This occurred because with an increase of temperature, adhesives enter high-elasticity instead of glassy states, which causes Al and adhesive surfaces to fail to bond closely; meanwhile, strength of adhesion produced by the mechanical interlock between the adhesive and the Al surface declines [54,55,56]. Therefore, fatigue cracks propagate to the Al–adhesive interface through the adhesive layer, and interfacial failure will eventually occur. At the same time, there were many fish-scale patterns along the direction of crack propagation, fracture surfaces of each layer were relatively smooth under low load level, and edges of each layer were slightly raised (Figure 9a,c), illustrating that plastic deformation occurred in the joint due to the high-elasticity-state adhesive layer.

Figure 9b,d,f present micro-failure morphologies of the joint under high load level (75% of failure load). For J-[0/90]_4s_ and J-[0/45/−45/90]_2s_, in this condition, the joints broke after fewer cycles, the temperature inside the joints was insufficient to degrade the interface bonding strength, and adhesive strength was less than interface bonding strength, causing cohesive failure. In addition, it was observed that some sharp particles existed on the fracture surface under the high load level, as shown in Figure 9b,d. This implies that high cyclic loads result in adhesive brittle fractures. For J-[45/−45]_4s,_ the matrix failed and the fracture surface was relatively smooth, and the fibres were pulled out. This phenomenon can be explained by the fact that the matrix of a CFRP adherend does not act as strong as the adhesive, and when a failure to the joint occurs, fibres are pulled out under great tension [57].

**Figure 11:**
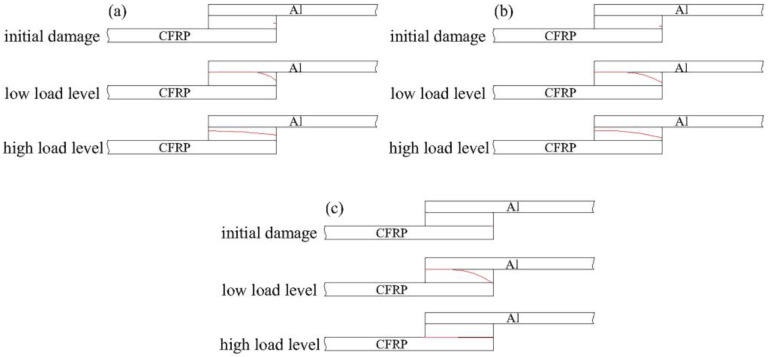
Schematic diagram of failure mechanism of joints: (**a**) [0/90]_4s_, (**b**) [0/45/−45/90]_2s_, and (**c**) [45/−45]_4s_.

As analysed above and shown in Figure 11, failure mechanisms of the joints were investigated, where the red line represents the damage path of the joint. From J-[0/90]_4s_ to J-[0/45/−45/90]_2s_, then to J-[45/−45]_4s_, the location of the initial damage gradually moved from the adhesive to the CFRP, owing to the decrease of interlaminar properties of the CFRP. Meanwhile, under high load level, the damage was less displaced in the lap thickness direction, and cohesive failure occurred with J-[0/90]_4s_ and J-[0/45/−45/90]_2s_, while delamination failure occurred with J-[45/−45]_4s_, since the matrix of CFRP bore the main load. Under low load, damage gradually moves from the initial damage location to the Al–adhesive interface due to a decrease in bonded strength of the Al–adhesive interface, and eventually, interface damage will occur. Therefore, matrix modification could be considered as a method to increase matrix strength and avoid delamination failure of J-[45/−45]_4s_ under high load level, and the Al–adhesive interface property should be heightened when the joints need to be used in service under low load level [58].

## 4. Conclusions

In this paper, three types of CFRP–Al single-lap adhesive joint (J-[0/90]_4s_, J-[0/45/−45/90]_2s_, and J-[45/−45]_4s_) were prepared for quasi-static and fatigue tests. Based on the Weibull statistical analysis method, effects of different stacking sequences on fatigue life of the joints were studied. Additionally, failure modes and microscopic fracture morphology were obtained in order to reveal the joint-failure mechanism under cyclic loads. Within limitations, some conclusions can be drawn, as follows:

(1) Stacking sequence can influence fracture mode of a joint under quasi-static loading. The brittle fracture of J-[0/90]_4s_ and J-[0/45/−45/90]_2s_ occurred due to 0° fibres undertaking the main load, while the ductile fracture of J-[45/−45]_4s_ occurred because the matrix bore the main load.
(2) The coefficient of variation of the joint varies depending on the stacking sequence. The stacking sequence can change the stability of the joint; the fatigue life of J-[45/−45]_4s_ fluctuated more than that of J-[0/45/−45/90]_2s_ and J-[0/90]_4s_.
(3) The initial failure location of the joint changes under influence of the stacking sequence. When the proportion of 45° plies in CFRP increased, the initial failure location gradually moved from the adhesive layer towards the CFRP due to the decline of fatigue performance of CFRP.
(4) Under high load levels, damage extends mainly from the initial failure location along the lap length direction and to a lesser extent in the lap thickness direction. Eventually, large-area cohesive failure occurred in joints in which the CFRP stacking sequences were [0/90]_4s_ and [0/45/−45/90]_2s_, and delamination failure occurred when the CFRP stacking sequence was [45/−45]_4s_.
(5) Under low load levels, all joints eventually suffered interfacial failure due to weakening of mechanical interlocking between the adhesive–Al interface, caused by the higher number of cycles; at this point, there was less effect of the stacking sequence on failure mode of the joint.

## Figures and Tables

**Table 1:** Material properties of USN15000/7901/RC33 [37].

Parameter	Symbol	Value
Longitudinal tensile modulus	*E_11_*/MPa	121,000
Transverse tensile modulus	*E_22_*/MPa	8600
Shear modulus	*G_1__2_*/MPa	3450
Poisson’s ratio	*μ_12_*	0.301
Density	*ρ*/kg·m^−3^	1570

**Table 2:** Material properties of Al 7075-T651 [38].

Parameter	Symbol	Value
Young’s modulus	*E*/MPa	71,700
Poisson’s ratio	*μ*	0.32
Density	*ρ*/kg·m^−3^	3000

**Table 3:** Specimen ID.

Specimen Full Name	Specimen ID
The joint with CFRP adherend stacking sequences of [0/90]_4s_	J-[0/90]_4s_
The joint with CFRP adherend stacking sequences of [0/45/−45/90]_2s_	J-[0/45/−45/90]_2s_
The joint with CFRP adherend stacking sequences of [45/−45]_4s_	J-[45/−45]_4s_

**Table 4:** Failure loads and displacements of the joints.

Specimen ID	Failure Load	Failure Displacement
J-[0/90]_4s_	5.93 kN	1.13 mm
J-[0/45/−45/90]_2s_	4.40 kN	1.47 mm
J-[45/−45]_4s_	3.52 kN	2.19 mm

**Table 5:** Fatigue life under different load levels with different stacking sequences.

Stacking Sequence	Load Level	Fatigue Life	lnln [1/*R*(*t*)]	ln(*t*)
[0/90]_4s_	100% (5.93 kN)	1	-	-
	75% (4.45 kN)	474	−1.753	6.161
		638	−0.717	6.458
		1026	−0.050	6.933
		1248	0.609	7.129
	55% (3.26 kN)	2442	−1.753	7.801
		3283	−0.717	8.097
		5235	−0.050	8.563
		7562	0.609	8.931
	35% (2.08 kN)	33,364	−1.753	10.415
		68,903	−0.717	11.140
		103,672	−0.050	11.549
		135,573	0.609	11.817
	25% (1.48 kN)	99,998	−1.753	11.513
		179,375	−0.717	12.097
		252,329	−0.050	12.620
		457,897	0.609	13.034
	15% (0.89 kN)	>1,000,000	-	-
[0/45/−45/90]_2s_	100% (4.40 kN)	1		
	75% (3.30 kN)	3706	−1.753	8.218
		4184	−0.717	8.339
		6464	−0.050	8.774
		10,801	0.609	9.287
	55% (2.42 kN)	4072	−1.753	8.312
		9015	−0.717	9.107
		10,837	−0.050	9.291
		20,441	0.609	9.925
	45% (1.98 kN)	15,661	−1.753	9.659
		37,542	−0.717	10.533
		52,921	-0.050	10.877
		103,162	0.609	11.544
	35% (1.54 kN)	30,780	−1.753	10.335
		81,592	−0.717	11.309
		215,581	−0.050	12.281
		258,906	0.609	12.574
	25% (1.10 kN)	>1,000,000		
	100% (3.52 kN)	1		
[45/−45]_4s_	75% (2.64 kN)	824	−1.753	6.714
		1015	−0.717	6.923
		1711	−0.050	7.445
		2716	0.609	7.907
	65% (2.29 kN)	4928	−1.753	8.503
		10,119	−0.717	9.222
		16,734	−0.050	9.725
		25,827	0.609	10.159
	55% (1.94 kN)	27,448	−1.753	10.220
		55,589	−0.717	10.926
		150,917	−0.050	11.924
		215,542	0.609	12.281
	45% (1.58 kN)	51,592	−1.753	10.851
		120,667	−0.717	11.701
		264,656	−0.050	12.486
		789,365	0.609	13.579
	35% (1.23 kN)	>1,000,000		

**Table 6:** Weibull parameters under different load levels.

Stacking Sequence	Load Level	*α*	*β*	*MTTF*	*CV*
[0/90]_4s_	75%	2.25	975.56	864	0.47
	55%	1.98	5372.48	4763	0.53
	35%	1.64	100,859.46	90,269	0.62
	25%	1.53	305,218.04	275,001	0.67
[0/45/−45/90]_2s_	75%	1.95	7201.55	6387	0.53
	55%	1.5	12,750.60	11,515	0.68
	45%	1.27	66,327.65	61,611	0.79
	35%	1.01	151,856.77	154,392	0.96
[45/−45]_4s_	75%	1.8	1869.33	1662	0.57
	65%	1.41	17,804.99	16,218	0.72
	55%	1.06	126,157.07	123,734	0.94
	45%	0.86	312,126.17	337,158	1.17

**Table 7:** Failure mode details of the joints.

Specimen ID	Load Level	Failure Mode
J-[0/90]_4s_	25%	cohesive failure (8%) + interfacial failure (92%)
	35%	cohesive failure (6%) + interfacial failure (94%)
	55%	cohesive failure (46%) + interfacial failure (54%)
	75%	cohesive failure (97%) + interfacial failure (3%)
	100%	cohesive failure (100%)
J-[0/45/−45/90]_2s_	35%	cohesive failure (20%) + interfacial failure (80%)
	45%	cohesive failure (34%) + interfacial failure (66%)
	55%	cohesive failure (62%) + interfacial failure (38%)
	75%	cohesive failure (85%) + interfacial failure (15%)
	100%	cohesive failure (98%) + slight fibre failure (2%)
J-[45/−45]_4s_	45%	cohesive failure (23%) + interfacial failure (77%)
	55%	cohesive failure (26%) + interfacial failure (74%)
	65%	cohesive failure (49%) + interfacial failure (51%)
	75%	delamination failure (100%)
	100%	delamination failure (100%)

## Data Availability

Not applicable.

## References

[1] Luan J.Z., Na J.X., Tan W., Mu W.L., Wang G.B., Gao Y. (2020). Comparative study on mechanical properties of aluminum alloy and BFRP single lap joints with hygrothermal aging. J. Adhes..

[2] Alam P., Robert C., Bradaigh C.M.O. (2018). Tidal turbine blade composites-A review on the effects of hygrothermal aging on the properties of CFRP. Compos. Part B Eng..

[3] Morgado M.A., Carbas R.J.C., Santos D.G.d., da Silva L.F.M. (2019). Strength of CFRP joints reinforced with adhesive layers. Int. J. Adhes. Adhes..

[4] Alireza D., Saeed R. (2021). Experimental and Numerical Study of Optimum Functionally Graded Aluminum/GFRP adhesive lap shear joints using Epoxy/CTBN. Int. J. Adhes. Adhes..

[5] Lokman G., Mohammed A., Ceyhun A., Sakir Y., Onuralp O.Y., Arslan, Hakan A.M. (2022). Optimum amount of CFRP for strengthening shear deficient reinforced concrete beams. Steel Compos. Struct..

[6] Aksoylu C., Yazman Ş., Özkılıç Y.O., Gemi L., Arslan M.H. (2020). Experimental analysis of reinforced concrete shear deficient beams with circular web openings strengthened by CFRP composite. Compos. Struct..

[7] Aksoylu C. (2021). Experimental analysis of shear deficient reinforced concrete beams strengthened by glass fiber strip composites and mechanical stitches. Steel Compos. Struct..

[8] Onuralp Ö.Y., Ceyhun A., Şakir Y., Lokman G., Hakan A.M. (2022). Behavior of CFRP-strengthened RC beams with circular web openings in shear zones: Numerical study. Structures.

[9] Hakan A.M., Şakir Y., Abed H.A., Ceyhun A., Onuralp Ö.Y., Lokman G. (2022). Shear strengthening of reinforced concrete T-beams with anchored and non-anchored CFRP fabrics. Structures.

[10] Gemi L., Aksoylu C., Yazman Ş., Özkılıç Y.O., Arslan M.H. (2019). Experimental investigation of shear capacity and damage analysis of thinned end prefabricated concrete purlins strengthened by CFRP composite. Compos. Struct..

[11] Ceyhun A. (2022). Shear strengthening of reinforced concrete beams with minimum CFRP and GFRP strips using different wrapping technics without anchoring application. Steel Compos. Struct..

[12] Sadeq A.H.A., Arife A., Ceyhun A., Musa H.A. (2022). Strengthening of shear-critical reinforced concrete T-beams with anchored and non-anchored GFRP fabrics applications. Structures.

[13] Onuralp Y.A., Sakir Y., Ceyhun A., Hakan A.M., Lokman G. (2021). Numerical investigation of the parameters influencing the behavior of dapped end prefabricated concrete purlins with and without CFRP strengthening. Constr. Build. Mater..

[14] Kgoete F.M., Popoola A.P.I., Fayomi O.S.I. (2019). Advancement in the application of alloys and composites in the manufacture of aircraft component: A review. J. Phys. Conf. Ser..

[15] Idris B., Yasir A., Jafar A., Gilles L., Nesar M. (2021). Fatigue crack growth in laser-treated adhesively bonded composite joints: An experimental examination. Int. J. Adhes. Adhes..

[16] Marques A.C., Mocanu A., Tomić N.Z., Balos S., Stammen E., Lundevall A., Abrahami S.T., Günther R., de Kok J.M.M., Teixeira de Freitas S. (2020). Review on adhesives and surface treatments for structural applications: Recent developments on sustainability and implementation for metal and composite substrates. Materials.

[17] Pramanik A., Basak A.K., Dong Y., Sarker P.K., Uddin M.S., Littlefair G., Dixit A.R., Chattopadhyaya S. (2017). Joining of carbon fibre reinforced polymer (CFRP) composites and aluminium alloys—A review. Compos. Part A Appl. Sci. Manuf..

[18] Banea M.D., Rosioara M., Carbas R.J.C., da Silva L.F.M. (2018). Multi-material adhesive joints for automotive industry. Compos. Part B Eng..

[19] Karataş M.A., Motorcu A.R., Gökkaya H. (2020). Optimization of machining parameters for kerf angle and roundness error in abrasive water jet drilling of CFRP composites with different fiber orientation angles. J. Braz. Soc. Mech. Sci. Eng..

[20] Jiang L.X., Xiao S.N., Dong D.W., Yang B., Chen D.D., Yang G.W., Zhu T., Wang M.M. (2022). Experimental study of bonded, bolted, and hybrid braided CFRP joints with different stacking sequences and lapping patterns. Thin-Walled Struct..

[21] Ozel A., Yazici B., Akpinar S., Aydin M.D., Temiz Ş. (2014). A study on the strength of adhesively bonded joints with different adherends. Compos. Part B Eng..

[22] Zhang Q., Cheng X.Q., Cheng Y.J., Li W.D., Hu R.W. (2019). Investigation of tensile behavior and influence factors of composite-to-metal 2D-scarf bonded joint. Eng. Struct..

[23] Machado J.J.M., Marques E.A.S., Barbosa A.Q., da Silva L.F.M. (2019). Effect of hygrothermal aging on the quasi-static behaviour of CFRP joints varying the overlap length. Compost. Struct..

[24] Fernández-Cañadas L.M., Ivañez I., Saez-Sonia S.E.J.B. (2019). Effect of adhesive thickness and overlap on the behavior of composite single-lap joints. Mech. Adv. Mater. Struct..

[25] Cai Y., An X.Z., Zou Q.C., Fu H.T., Yang X.H., Zhang H. (2021). Mechanical properties and failure mechanisms of composite laminates with classical fabric stacking patterns. J. Mater. Sci..

[26] He Z.K., Luo Q.T., Li Q., Zheng G., Sun G.Y. (2022). Fatigue behavior of CFRP/Al adhesive joints-Failure mechanisms study using digital image correlation (DIC) technique. Thin-Walled Struct..

[27] Federal Aviation Administration (2017). Federal Aviation Regulations FAR 25 Airworthiness Standards: Transport Category Airplanes.

[28] Federal Aviation Administration (2010). Advisory Circular AC 25.571-1D: Damage Tolerance and Fatigue Evaluation of Structures.

[29] Budhe S., Banea M.D., de Barros S., da Silva L.F.M. (2017). An updated review of adhesively bonded joints in composite materials. Int. J. Adhes. Adhes..

[30] Wahab M.M.A., Bartolomé J.F., Chicot D., Lowther J.E. (2012). Fatigue in Adhesively Bonded Joints: A Review. ISRN Mater. Sci..

[31] Mariam M., Afendi M., Majid M.S.A., Ridzuan M.J.M., Gibson A.G. (2018). Tensile and fatigue properties of single lap joints of aluminium alloy/glass fibre reinforced composites fabricated with different joining methods. Compost. Struct..

[32] Azari S., Ameli A., Papini M., Spelt J.K. (2013). Adherend thickness influence on fatigue behavior and fatigue failure prediction of adhesively bonded joints. Compos. Part A Appl. Sci. Manuf..

[33] Kara E., Kurşun A., Haboğlu M.R., Enginsoy H.M., Aykul H. (2015). Fatigue behavior of adhesively bonded glass fiber reinforced plastic composites with different overlap lengths. Proc. Inst. Mech. Eng. Part C J. Mech. Eng. Sci..

[34] Shin K.C., Lee J.J. (2010). Effects of bond parameters on fatigue characteristics of a cocured double lap joint subjected to cyclic tensile loads. J. Adhes..

[35] Hedayati R., Khouzani S.G., Jahanbakhshi M. (2015). Investigation of debonding propagation in aluminum/composite joints under fatigue loading. J. Adhes. Sci. Technol..

[36] Meneghetti G., Quaresimin M., Ricotta M. (2012). Damage mechanisms in composite bonded joints under fatigue loading. Compos. Part B Eng..

[37] Tian T.C., Fu J., Ju Y.Z. (2022). Experimental study on failure mechanism of CFRP-to-aluminium single-lap adhesive joints under tension after out-of-plane pre-impact. J. Adhes..

[38] Zhao T., Jiang Y. (2007). Fatigue of 7075-T651 aluminum alloy. Int. J. Fatigue.

[39] (2020). Standard Test Method for Fatigue Properties of Adhesives in Shear by Tension Loading (Metal/Metal).

[40] (2014). Standard Test Method for Lap Shear Adhesion for Fiber Reinforced Plastic (FRP) Bonding.

[41] Çavdar S., Teutenberg D., Meschut G., Wulf A., Hesebeck O., Brede M., Mayer B. (2019). Stress-based fatigue life prediction of adhesively bonded hybrid hyperelastic joints under multiaxial stress conditions. Int. J. Adhes. Adhes..

[42] Xu X.X., Crocombe A.D., Smith P.A. (2006). Fatigue Crack Growth Rates in Adhesive Joints Tested at Different Frequencies. J. Adhes..

[43] Liu X.L., Zheng G., Luo Q.T., Qing L., Sun G.Y. (2021). Fatigue behavior of carbon fibre reinforced plastic and aluminum single lap adhesive joints after the transverse pre-impact. Int. J. Fatigue.

[44] Datla N.V., Ameli A., Azari S., Papini M., Spelt J.K. (2012). Effects of hygrothermal aging on the fatigue behavior of two toughened epoxy adhesives. Eng. Fract. Mech..

[45] Haidyrah A.S., Newkirk J.W., Castaño C.H. (2016). Weibull statistical analysis of Krouse type bending fatigue of nuclear materials. J. Nucl. Mater..

[46] Naresh K., Shankar K., Velmurugan R. (2018). Reliability analysis of tensile strengths using Weibull distribution in glass/epoxy and carbon/epoxy composites. Compos. Part B Eng..

[47] Hao J., Tong L., Li G.Y., Zhang X., Cui J.J. (2017). Fatigue life assessment of electromagnetic riveted carbon fiber reinforce plastic/aluminum alloy lap joints using Weibull distribution. Int. J. Fatigue.

[48] Mollón V., Bonhomme J., Viña J., Argüelles A., Fernández-Canteli A. (2012). Influence of the principal tensile stresses on delamination fracture mechanisms and their associated morphology for different loading modes in carbon/epoxy composites. Compos. Part B Eng..

[49] Davidson P., Waas A.M. (2017). The effects of defects on the compressive response of thick carbon composites: An experimental and computational study. Compos. Struct..

[50] Modi V., Singh K.K., Shrivastava R. (2020). Effect of Stacking Sequence on Interlaminar Shear Strength of Multidirectional GFRP Laminates. Mater. Today.

[51] Gudladt H.J., Frömmel S.T. (2019). Fatigue and fracture behavior of adhesive-bonded structures in the light of the surface morphology. Int. J. Adhes. Adhes..

[52] Hull D. (1999). Fractography, Observing, Measuring and Interpreting Fracture Surface Topography.

[53] Wei T., Na J.X., Wang G.B., Chen H.L., Meng H. (2021). Effect of temperature on the fatigue performance and failure mechanism of a flexible adhesive butt joint. J. Adhes..

[54] Yoon P.S., Jong C.W., Hoon C.C., Soap C.H. (2018). The effect of curing temperature on thermal, physical and mechanical characteristics of two types of adhesives for aerospace structures. J. Adhes. Sci. Technol..

[55] Reis J.M.L., Amorim F.C., da Silva A.H.M.F.T., da Costa H.S.M. (2015). Influence of temperature on the behavior of DGEBA (bisphenol A diglycidyl ether) epoxy adhesive. Int. J. Adhes. Adhes..

[56] Flinn R.A., Trojan P.K. (1995). Engineering Materials and Their Applications.

[57] Demiral M., Kadioglu F. (2018). Failure behaviour of the adhesive layer and angle ply composite adherends in single lap joints: A numerical study. Int. J. Adhes. Adhes..

[58] Fan X.M., Yin X.W. (2018). Progress in research and development on matrix modification of continuous fiber-reinforced silicon carbide matrix composites. Adv. Compos. Hybrid Mater..

